# Stroke Associated With Recent Mycoplasma Pneumoniae Infection: A Systematic Review of Clinical Features and Presumed Pathophysiological Mechanisms

**DOI:** 10.3389/fneur.2018.01109

**Published:** 2018-12-21

**Authors:** Nicolas Mélé, Guillaume Turc

**Affiliations:** ^1^Service de Neurologie, Centre Hospitalier Sainte-Anne, Paris, France; ^2^Université Paris Descartes, Paris, France; ^3^INSERM UMR 894, Paris, France; ^4^DHU Neurovasc, Paris, France

**Keywords:** Mycoplasma pneumoniae, stroke, hypercoagulability, vasculitis, infection

## Abstract

**Introduction:** An association between Mycoplasma pneumoniae (MP) infection and stroke has been described, especially in children. However, current knowledge on this rare potential cause of stroke is scant. The purpose of this systematic review of all published cases was to help better understand the relationships between recent MP infection and ischemic stroke on a clinical, radiological and pathophysiological perspective.

**Material and Methods:** A PubMed and Embase search was performed in September 2018 to identify all published cases of stroke occurring within 4 weeks after MP infection.

**Results:** Twenty-eight patients with ischemic stroke associated with MP infection were identified. Median age was 8 years (range: neonate to 57). The middle cerebral artery territory was involved in 25 (89%) patients. Fifteen (54%) patients had at least one arterial occlusion. Elevated D-dimer and/or fibrinogen was reported in 8 (29%) patients. Four patients had transient anticardiolipin IgM antibodies. Cerebrospinal fluid analysis showed pleocytosis in 7/20 (35%) patients (median: 19 leucocytes/μL, range: 10 to 63) and MP PCR was positive in 3/8 (38%) patients. The etiological work-up was considered inconclusive in 25 (89%) patients. Three (11%) patients died during follow-up, all of early respiratory deterioration. Neurological functional outcome was good in 22/27 (81%) patients.

**Conclusion:** The association between MP infection and ischemic stroke in children and young adults is rare. Underlying pathogenesis might include hypercoagulability and vasculitis. Most patients achieve a favorable recovery. Whether MP infection could be a long-term risk factor for stroke by promoting atherosclerosis is uncertain and deserves further investigation.

## Introduction

Mycoplasma pneumoniae (MP), a bacterium which belongs to the class *Mollicutes*, is responsible for up to 40% of community-acquired pneumonia, and affects most frequently young children (1, 2). Pulmonary infections due to MP are usually mild and self-limited, and many are asymptomatic (3, 4). Central nervous system (CNS) involvement is the most commonly reported extra-pulmonary feature, occurring in 1 to 10% of patients with serologically confirmed MP infections requiring hospitalization (5, 6). CNS manifestation typically include meningo-encephalitis, optic neuritis or transverse myelitis, and cases of stroke occurring shortly after MP infection have been reported, mainly in young patients (6, 7). However, due to the rarity of this association, only case reports have been published so far. Therefore, our current knowledge of the underlying pathogenesis, clinical features, paraclinical findings, and outcome of stroke occurring after MP infection remains poor. A large prospective study suggested that patients with recent MP infection had a higher risk of subsequent ischemic stroke than comorbidity-matched controls over a 5-years follow-up period (8). Those findings suggest that MP infection could increase the risk of ischemic stroke even over a long-term period, which might be due to different mechanisms (9, 10).

The purpose of this systematic review of the literature was to help improve current knowledge on the relationships between recent MP infection and ischemic stroke on clinical, radiological, and pathophysiological perspectives.

## Methods

Studies or case reports were eligible for the present review if they: (1) included patient with ischemic or hemorrhagic stroke proven by computerized tomography (CT-scan) or magnetic resonance imaging (MRI), regardless of vascular territory or presumed cause; (2) with recent (less than 4 weeks) MP infection proven by serology, culture or polymerase chain reaction (PCR); (3) and featured data on age, sex, neurological symptoms and follow-up.

**Figure 1:**
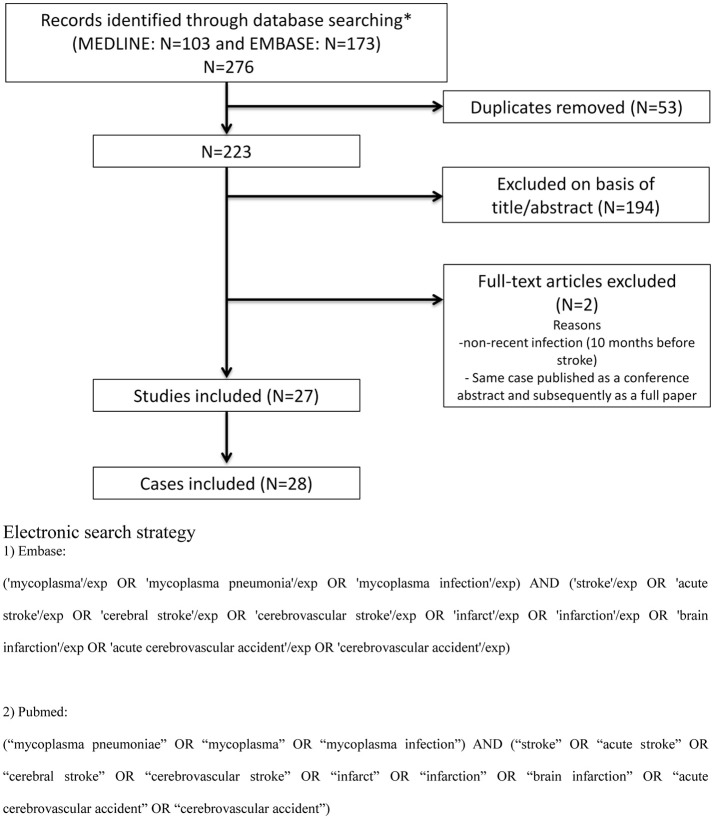
PRISMA diagram and electronic search strategy.

We searched Medline/PubMed and Embase for studies published in English or French between 01/01/1980 and 21/09/2018, using predefined search terms (Figure 1). We also hand-searched the reference lists of all included articles and any relevant review articles. A flow diagram of studies screened, assessed for eligibility and included was prepared according to Preferred Reporting Items for Systematic reviews and Meta- Analyses (PRISMA) guidelines (Figure 1) (11).

For each included publication, the following data were extracted by one reader (NM): age, sex, type, and severity of respiratory symptoms, time between the onset of potential respiratory symptoms and stroke, stroke territory, type, and severity of stroke, type of brain imaging (MRI or CT scan), type and results of stroke etiological work-up, results of blood and cerebrospinal fluid (CSF) analysis, neurological outcome, and length of follow-up. Based on available information in each article, we also estimated the modified Rankin Scale score at the end of follow-up (12). A favorable neurological outcome was defined as an estimated modified Rankin Scale score ≤ 2. Only descriptive statistics were performed. Continuous variables were expressed as median (range), and categorical variables as numbers and percentages.

## Results

Among 223 studies and records identified through database searching, 27 studies met the inclusion criteria (Figure 1), reporting a total of 28 patients who had an ischemic stroke within 4 weeks of MP infection (7, 13–38). No patient with intracerebral hemorrhage was identified. Patient description is summarized in Table 1. Median age was 8 years (range: neonate to 57), and 19 (67%) patients were aged 15 or younger. Fifteen (54%) patients were female. MP infection was proven by serology in all patients using various methods of testing including ELISA, complement fixation, cold agglutinin assay or immunoblot. Twenty-four (86%) patients had recent upper respiratory tract infection (including severe pneumonia in 5 patients), while the 4 remaining patients were diagnosed with recent MP infection in spite of having no respiratory symptoms. The median interval between the onset of respiratory symptoms and stroke was 9 days (range: 2 to 21). No patient had extrapulmonary symptoms other than stroke. Brain magnetic resonance imaging (MRI) was performed in 18 (64%) patients. Twenty-five (89%) patients had a stroke in the territory of the middle cerebral artery (MCA), and 4 (14%) in the posterior circulation. All patients underwent an evaluation of intracranial vessels, by duplex ultrasonography, computed tomography angiography, magnetic resonance angiography, and/or conventional arteriography. Fifteen (54%) patients had at least one arterial occlusion, most frequently involving the MCA. Occlusion of the posterior circulation was observed in only 4 patients (21, 26, 27, 33). Altogether, 11 (39%) patients had a large vessel occlusion (internal carotid artery, proximal MCA or basilar artery occlusion). Results of extracranial cervical artery imaging were explicitely reported in 13 (46%) patients and showed arterial occlusion in 5 patients, non-occlusive carotid thrombus in 2 patients, and aneurysm in the subclavian artery and aortic arch in one patient. Nineteen (66%) patients underwent cardiac work-up, including transthoracic echocardiography (*n* = 18), transesophageal echocardiography (*n* = 4) and holter ECG monitoring (*n* = 3). Cardiac evaluation was normal in all cases except in two patients with patent foramen ovale (29, 38). Increased fibrinogen and/or elevated D-dimer were reported in 8 (29%) patients (Table 1). There were 4/13 (31%) patients with positive anticardiolipin IgM antibodies, two of whom had prolonged partial thromboplastin time (20, 25, 29, 38). One of them had positive lupus anticoagulant and positive antinuclear antibodies (1:240) (20), while another one had positive anti beta2 GP1 antibodies and hemolytic anemia (38). At the end of follow-up, no patient was reported to have persistant anticardiolipin antibodies, lupus anticoagulant or prolonged partial thromboplastin time. Regarding other laboratory tests, one patient had sickle cell trait and another had a mutation in the methylenetetrahydrofolate reductase gene (21, 26). CSF analysis showed pleocytosis (≥10 leucocytes/μL) in 7/20 (35%) patients (median: 19 leucocytes/μL, range: 10 to 63) and MP PCR was positive in 3/8 (38%) patients.

**Table 1:** Patient description.

**References**	**Age/sex**	**Respiratory Illness^**a**^**	**Stroke territory/presence of occlusion**	**Modality of MP diagnosis^‡^**	**CSF analysis**	Laboratory tests for hypercoagulability^*^	**Follow-up/estimated mRS**
Parker et al. (13)	8y/♀	6 days (pneumonia)	Left MCA/no occlusion	CF	Normal, culture (−)	Normal	2 months/mRS 0
Nakahata et al. (14)	4y/♂	9 days (pneumonia)	Left MCA/parietal artery occlusion	No data	63 leucocytes/Ml	Normal	No data/mRS 0
Dowd et al. (15)	31y/♀	3 weeks (pneumonia)	Right MCA/no occlusion	No data	No data	No data; presence of cold agglutinins	No data/mRS 2
Mulder et al. (16)	30y/♀	7 days (pneumonia)	Left MCA/proximal MCA occlusion	CF	Normal	↑fibrinogen, ↑FDP	3 years/mRS 2
Visudhiphan et al. (17)	12y/♀	10 days (upper respiratory tract infection)	Left ICA/ICA occlusion	CF	Normal	Normal	1 year/mRS 2
Fu et al. (18)	5y/♀	10 days (upper respiratory tract infection)	Left MCA/proximal MCA occlusion	CF	Normal	↑fibrinogen, ↑D-dimer	8 months/mRS 1
Socan et al. (19)	28y/♀	6 days (productive cough)	Right MCA/distal MCA occlusion	I	Normal, culture (+) and PCR (+)	Normal	6 months/mRS 1
Padovan et al. (20)	36y/♀	8 days (upper respiratory tract infection)	Right MCA/No occlusion ?	CAA	Normal, serology (+) and PCR (+)	IgM (+) of aCL^†^, ↑activated partial thromboplastin time, positive lupus anticoagulant, ↑D-dimer	6 months/mRS 3
Antachopoulos et al. (21)	8y/♂	14 days (pneumonia)	Left PCA/proximal PCA occlusion or severe stenosis	I	Normal	Sickle cell trait	6 months/mRS 1
Ovetchkine et al. (22)	8y/♂	No respiratory symptoms	Right MCA/no occlusion, multiple stenosis in the MCA territory	E	10 leucocytes/μL, culture (−) and PCR (−)	Normal	1 year/mRS 0
Idbaih et al. (23)	35 y/♀	3 weeks (pneumonia)	Right ACA and MCA/no occlusion, ICA mural thrombus	No data	No data	No data	No data/mRS 0
Leonardi et al. (24)	6y/♂	3 days (upper respiratory tract infection)	Left MCA/no occlusion	CAA	Normal, serology (+), culture (−) and PCR (−)	Normal	1 week/mRS 6
Leonardi et al. (24)	5y/♀	14 days (upper respiratory tract infection)	Left MCA/proximal MCA occlusion	CAA	Normal, serology (+), culture (−)	Normal	6 months/mRS 0
Tanir et al. (25)	7y/♀	10 days (severe pneumonia)	Left MCA/ICA occlusion	E	Normal	IgM (+) of aCL^†^ and aPL; thrombocytopenia (61 G/L)	6 months/mRS 0
Ryu et al. (26)	13y/♂	No respiratory symptoms	Vertebrobasilar/Right vertebral and basilar artery occlusion	E	19 leucocytes/μL	MTHFR mutant type	1 month/mRS 0
Lee et al. (27)	4y/♂	9 days (severe pneumonia)	Bilateral Fronto-parieto-occipital (watershed) infarction/bilateral ICA and bilateral vertebral artery occlusion	CAA	Normal, PCR (−)	↑fibrinogen, ↑D-dimer, ↑FDP	3 months/mRS 6
Siclari et al. (28)	40y/♀	7 days (pneumonia)	Left MCA/ICA and MCA occlusion; endoluminal thrombi in both common carotid arteries	CAA	No data	Normal	1 month/mRS 4
Senda et al. (29)	21y/♂	7 days (upper respiratory tract infection)	Left MCA/proximal MCA occlusion	CAA	Normal	IgM (+) of aCL^†^, ↑prothrombin time, ↑activated partial thromboplastin time, ↑fibrinogen, ↑D-dimer, ↑thrombin-antithrombin III-complex, ↓protein C	6 months/mRS 1
Garcia et al. (30)	13y/♀	5 days (severe pneumonia)	Right ACA-MCA and left MCA/No data	No data	No data	No data	No data/mRS 6
Kim et al. (7)	3y/♀	7 days (pneumonia)	Right MCA/No occlusion	E	13 leucocytes/μL, culture (−)	↓protein S, ↑fibrinogen	1 month/mRS 0
Bashiri et al. (31)	10y/♂	2 weeks (pneumonia)	Left anterior choroidal artery/No occlusion	No data	No data	Normal	No data/mRS 1
Bao et al. (32)	8y/♂	2 weeks (severe pneumonia)	Left MCA/Central retinal artery occlusion	E	40 leucocytes/μL, culture (−) and PCR (−)	No data	2 months/mRS 1
Kunzmann et al. (36)	0y/♂	No respiratory symptoms	Bilateral MCA/No occlusion	CSF	17 leucocytes/μL, PCR (+)	No data	3 months/mRS 0
Kang et al. (37)	5y/♀	6 days (pneumonia)	Right MCA/proximal MCA occlusion	E	Normal, PCR (−)	↑fibrinogen, ↑D-dimer	1 month/mRS 2
Benghanem et al. (38)	57y/♀	2 weeks (pneumonia)	Bilateral punctiform infarcts/No occlusion	E	No data	Positive aCL and anti beta2 GP1 Ab; Hemolytic anemia	No data
Garcia Tirado et al. (33)	6y/♂	2 days (productive cough)	Both PCA and left MCA/PCA occlusion	No data	No data	Normal	1 month/mRS 1
Sarathchandran et al. (34)	39y/♂	No respiratory symptoms	Left MCA/No occlusion; subclavean and aortic arch aneurysms	No data	58 leucocytes/μL, culture (−)	No data	3 months/mRS 0
Jin et al. (35)	7y/♂	2 weeks (severe pneumonia)	Left MCA/ICA and MCA occlusion	No data	No data	↑D-dimer	6 months/mRS 2

*Ab, antibody; aCL, anticardiolipin antibody; aPL, antiphospholipid antibody; CCA, common carotid arteries; CSF, cerebrospinal fluid; ICA, internal carotid artery; IgM, immunoglobulin M; FDP, fibrin degradation product; MCA, middle cerebral artery; MTHFR, methylenetetrahydrofolate reductase gene; PCA, posterior cerebral artery; PCR, polymerase chain reaction; WBC, white blood cell*.

‡ *CAA, cold agglutinin assay; CF, complement fixation; E, ELISA; I, immunoblot; Cerebrospinal fluid*.

* *Any laboratory abnormalities suggestive of hypercoagulability*.

† *Anticardiolipin IgM antibodies: 92 U/ml, normal < 15 U/ml (23); 16.1 RU/ml, normal < 12 RU/ml (28); 220 MPL, normal < 30 (32)*.

a *This column depicts the time between the onset of respiratory illness and the onset of ischemic stroke*.

The etiological work-up was considered inconclusive in 25 (89%) patients. Two patients had a patent foramen ovale associated with proximal deep vein thrombosis or pulmonary embolism, suggesting a paradoxical embolism (29, 38). Another patient was diagnosed with vasculitis based on multifocal narrowing with areas of localized dilatation on digitally subtracted angiography and evidence of CSF pleocytosis (22). Localized vasculitis and hypercoagulability were hypothesized in many publications.

Antibiotic treatment was reported in 26 (93%) patients, most frequently involving intravenous macrolides (19 patients). Nine patients were treated with aspirin, 5 with heparin, 8 with corticosteroids and 3 with intravenous immunoglobulins. No patient received long-term treatment with aspirin or anticoagulation. Duration of follow-up was mentioned in 22 (79%) patients (median: 4.5 months, range: 1 week to 3 years). Three (11%) patients died due to respiratory failure. Neurological functional outcome in survivors was good (estimated modified Rankin Scale score ≤ 2) in 22/27 (81%) patients (median estimated mRS: 1, range: 0 to 6). Neurological recovery was complete for 10/27 (37%) patients. No patient experienced a recurrent stroke during follow-up.

## Discussion

In this systematic review of the literature, we have analyzed 28 patients with ischemic stroke associated with recent MP infection, published as single or dual case reports. The median interval between the onset of respiratory symptoms and stroke was 9 days. No case of intracerebral hemorrhage was observed. All patients but one were children or young adults, which may reflect the fact that MP-related pneumonia affect mostly young people, (1, 2) but also that more extensive biological work-up is often performed when stroke occurs in a young patient (39). In many reports, numerous alternative infections were screened, especially among patients with no preceding respiratory symptoms (e.g., Herpes simplex virus, Varicella zoster virus, Epstein–Barr virus, Chlamydia species, Bartonella henselae, Borrelia burgdorferi). None of these investigations were reported to be positive. Provided that MP is one of the most common pathogens of respiratory tract infection in children, the small number of reported cases of stroke occurring after MP infection confirms that this association is very rare. Furthermore, at the individual patient level, whether MP infection was directly involved in the pathophysiological cascade of stroke, represented a risk factor or was simply an incidental finding is difficult to determine. Indeed, a clearly identified cause of stroke was only identified in 3 patients, one with cerebral vasculitis and two with probable paradoxical embolism (proximal deep vein thrombosis or pulmonary embolism associated with a patent foramen ovale). These cases illustrate two important mechanisms which have been suggested to link MP infection and stroke, namely vasculitis and hypercoagulabity (5).

After colonizing the oropharynx, MP can invade the bloodstream and subsequently the CNS, similarly to other pathogens. Evidence of direct invasion has been demonstrated by the growth of live MP organisms in culture media after direct sampling of CNS tissue or cerebrospinal fluid in cases of acute encephalitis or stroke (19, 20, 40, 41). The vascular transfer of the organism itself could locally affect the vascular walls of the cerebral circulation by the induction of cytokines and chemokines such as tumor necrosis factor-α and interleukin-8 (42–44). This may cause local vasculitis, thrombotic vascular occlusion, or both, even in the absence of a systemic hypercoagulable state. Components of MP organisms may also be transported across the blood-brain barrier and lead to a consecutive immunologic reaction within the CNS (40, 41, 45). The occurrence of focal vasculitis is supported by the finding that mycoplasma-like structures have been found in granulomatous vasculitis of small branches of cerebral arteries (46). Conversely, molecular studies failed to detect the pathogen in the CSF in two other cases of vasculitis without stroke (47, 48), raising the hypothesis that a mechanism other than direct vascular invasion by MP could also be involved. Indeed, late-onset vasculopathy occurring about 2–3 weeks after the respiratory episode might be better explained by immune complex mediated injury. Through this mechanism, MP-derived antigenic elements that are bound to blood macrophages or monocytes could initiate complex immune reactions and subsequent vasculitis (22, 47, 49).

Hypercoagulability induced by MP is another mechanism that may lead to stroke occurrence and is thought be related to surface proteins and chemical mediators produced by MP (50, 51). *In vitro* experimental studies indeed suggest that procoagulant activity (tissue factor-like activity) can be induced by lipoglycans of MP via human mononuclear cells (52). Leukocyte activation under septic state may also induce the release of tumor necrosis factor and interleukin-1, which may in turn induce endothelial organ activation, and likely alter the normal anticoagulant state of the endothelium tissue. A hypercoagulable state associated with unbalanced endothelial function would facilitate intra-vascular coagulation in the venous or arterial beds (53, 54). Subsequent thromboembolism may lead to cerebral artery occlusion, which was observed in approximately half of the patients in the present review, sometimes in more than one territory. Of note, elevated levels of fibrin, D-dimer or fibrinogen were reported in 8 patients, which is consistent with a recent study showing a change in blood coagulation parameters, including plasma D-dimer and fibrinogen levels, in children with recent MP pneumonia infection (55).

Like many other infectious agents, MP has been associated with the presence of transient anticardiolipin antibodies (56). However, IgM anticardiolipin antibodies were documented in only 4 patients in the present review, and became negative during follow-up. These patients did not meet the criteria for antiphospholipid antibody syndrome due to a lack of persistent anticardiolipin antibodies or lupus anticoagulant (57). In contrast to phospholipid antibody syndrome, patients with anticardiolipin antibodies induced by infection are generally not thought to be at increased risk of thrombosis, because such patients exhibit the characteristics of natural autoantibodies rather than those of the pathogenic autoantibodies that are found in patients with systemic lupus erythematosus (58, 59).

Clinical features of stroke associated with MP were non-specific, and 9 out of 10 patients had a stroke in the middle cerebral artery territory, consistent with characteristics of ischemic stroke in childhood (60). Half of the patients had at least one occlusion, most frequently concerning the proximal MCA or terminal internal carotid artery (ICA). CSF pleocytosis was rarely observed and slight, as was the identification of MP in the CSF. However, CSF evaluation was missing in many patients, limiting the interpretation of these results. Prognosis of stroke related to recent MP infection was good in a majority of patients. However, this condition can lead to death related to respiratory failure at the early phase, irrespective of stroke severity. Once passed the early stage, the prognosis was good, even in case of large cerebral infarction. Almost all patients were treated with antibiotics, either before or after stroke onset. The available data does not allow to draw a conclusion on the respective impact of antithrombotics, antibiotic therapy, corticosteroids or their combination on neurological outcome.

No stroke recurrence was documented, but the median follow-up was only 4.5 months. By contrast, a large population study in Taiwan showed that patients who had a MP infection seem to be, in the long term, at higher risk of ischemic stroke than comorbidity-matched controls, even after adjustment for potential confounding factors (8). These findings suggest that MP infection could increase the risk of ischemic stroke even over a long-term period. Persistent inflammatory processes and hypercoagulable state after MP infection might play a role but other mechanisms have been suggested. Indeed, MP has been found in atherosclerotic plaques (9), and increased serum antibody of MP in patients with coronary artery disease has been reported (10), which raised the hypothesis that MP could promote atherosclerosis. However, although MP infection may be independently associated with the risk of subsequent ischemic stroke development (8), studies of the seroprevalence of MP in stroke patients and controls led to conflicting results (61, 62). Further investigations are needed to confirm that MP infection is an independent risk factor for stroke in the long-term and to better understand the relationships between MP infection and atherosclerosis.

Our review has several limitations. First, stroke etiological workup was suboptimal in many patients, with extracranial artery imaging and echocardiography performed in a minority of patients. It is therefore possible that alternative causes of stroke in young patients, such as cervical artery dissection, for which recent infection could be a risk factor (63), or presence of a patent foramen ovale (64), may have been overlooked. Second, detailed information was often lacking with regard to results of CSF analysis, therapeutic management and follow-up. The quality of future case reports may increase following the publication of the Consensus-based Clinical Case Reporting Guideline Development (65). Third, the number of published cases was relatively small and there was an important variability among patients regarding diagnostic procedures, stroke etiological work-up and therapeutics. This was expected because the literature mostly consists of reports of single patients managed in different centers, but it may nonetheless limit the comparability of patients and the validity of the collective findings. However, the infrequency of stroke in patients with MP infection would hamper the feasibility of a dedicated prospective study. Finally, publication bias toward cases with particular findings or more severe forms of disease cannot be excluded.

## Conclusion

Clinicians should be aware of the potential risk of cerebral ischemic stroke in children and young adults with MP respiratory tract infection, even though this association is very rare. Stroke associated with MP infection has good prognosis. The pathogenesis of stroke associated with MP is probably multifactorial, and may include immune hypercoagulable state and vasculitis. Whether MP infection could be a long-term risk factor for stroke by promoting atherosclerosis deserves further investigation.

## Data Availability Statement

All datasets generated and analyzed for this study are included in the manuscript.

## Author Contributions

GT and NM: study concept and design; NM: acquisition of data; NM and GT: analysis and interpretation of data: NM: drafting of manuscript; GT: manuscript revision for important intellectual content.

### Conflict of Interest Statement

The authors declare that the research was conducted in the absence of any commercial or financial relationships that could be construed as a potential conflict of interest.
